# Efficacy and Safety of Dual and Triple Glucagon-Like Peptide-1-Based Polyagonists in Metabolic Dysfunction-Associated Steatotic Liver Disease and Steatohepatitis: A Systematic Review and Meta-Analysis

**DOI:** 10.7759/cureus.111728

**Published:** 2026-06-29

**Authors:** Rogelio Santos Solis, Armando A Baeza-Zapata, Juan P Negrete-Najar

**Affiliations:** 1 Internal Medicine, Hospital General Regional No. 1, Instituto Mexicano del Seguro Social, Chihuahua, MEX; 2 Gastroenterology and Hepatology, Hospital Angeles Chihuahua, Chihuahua, MEX; 3 Geriatrics, Instituto Nacional de Geriatría, Mexico City, MEX

**Keywords:** glp-1 based polyagonist, glp-1/gip receptor agonists, liver fibrosis, metabolic dysfunction-associated steatohepatitis (mash), metabolic dysfunction-associated steatotic liver disease

## Abstract

Metabolic dysfunction-associated steatotic liver disease (MASLD) and its inflammatory form, metabolic dysfunction-associated steatohepatitis (MASH), are strongly linked to obesity, insulin resistance, type 2 diabetes, and progressive liver fibrosis. Dual and triple glucagon-like peptide-1 (GLP-1)-based polyagonists may provide complementary hepatic and metabolic benefits, but their overall efficacy and safety have not been fully established. This systematic review and meta-analysis evaluated randomized controlled trials comparing dual or triple GLP-1-based polyagonists with placebo in adults with MASLD or MASH. PubMed, the Cochrane Central Register of Controlled Trials, ScienceDirect, and Google Scholar were searched from inception through May 19, 2026. Dichotomous outcomes were pooled as risk ratios (RRs), and continuous outcomes were analyzed as mean differences (MDs), using random-effects models. Risk of bias was assessed using the Cochrane Risk of Bias 2 tool. Six randomized controlled trials involving 961 participants were included. The included GLP-1-based polyagonists-tirzepatide, survodutide, pemvidutide, retatrutide, and cotadutide-significantly increased MASH resolution or histological improvement without worsening of fibrosis (RR 3.32, 95% CI 2.28-4.84; I² = 20%) and fibrosis improvement without worsening of MASH (RR 1.49, 95% CI 1.15-1.94; I² = 0%). Treatment also produced a greater relative reduction in liver fat content (MD -44.60 percentage points, 95% CI -51.17 to -38.04; I² = 0%) and increased the likelihood of achieving at least a 30% relative reduction in liver fat by magnetic resonance imaging-proton density fat fraction (RR 4.71, 95% CI 3.04-7.30; I² = 22%). Body weight was significantly reduced, although heterogeneity was considerable (MD -9.14 percentage points, 95% CI -17.38 to -0.91; I² = 98%). Nausea, diarrhea, and vomiting were more frequent with polyagonists, but no statistically significant differences were observed in serious adverse events or adverse events leading to treatment discontinuation. In conclusion, dual and triple GLP-1-based polyagonists improve histological and imaging-based hepatic outcomes in MASLD and MASH and may simultaneously address underlying metabolic dysfunction. However, gastrointestinal intolerance, pharmacological heterogeneity, the small number of trials, and limited follow-up warrant cautious interpretation. Larger phase 3 trials are needed to clarify drug-specific efficacy, long-term safety, and effects on liver-related and cardiovascular outcomes.

## Introduction and background

Metabolic dysfunction-associated steatotic liver disease (MASLD), the newly adopted term that has largely superseded nonalcoholic fatty liver disease (NAFLD) in the updated nomenclature, is one of the most common chronic liver disorders globally and an increasingly important public health concern [1-6]. ​​​The updated multisociety nomenclature defines MASLD by hepatic steatosis accompanied by one or more cardiometabolic risk factors and replaces the term "non-alcoholic steatohepatitis" (NASH) with "metabolic dysfunction-associated steatohepatitis" (MASH) [1]. This terminology emphasizes the close relationship between steatotic liver disease and cardiometabolic conditions such as excess adiposity, impaired insulin sensitivity, diabetes, abnormal lipid metabolism, elevated blood pressure, and cardiovascular complications rather than defining the condition primarily by the exclusion of alcohol consumption [1-5].

Contemporary clinical guidelines recognize MASLD as a multisystem metabolic disorder requiring coordinated management of hepatic and extrahepatic risk [2-5]. Guidance jointly issued by the European associations for liver disease, diabetes, and obesity recommends targeted case-finding for MASLD with clinically significant fibrosis among individuals with type 2 diabetes, obesity accompanied by additional metabolic risk factors, persistently elevated liver enzyme levels, or imaging findings consistent with hepatic steatosis [2]. Likewise, the American Association for the Study of Liver Diseases (AASLD) advises a sequential noninvasive risk assessment, beginning with readily available fibrosis scores and, when indicated, followed by elastography or specialized serum biomarkers [3]. The American Association of Clinical Endocrinology (AACE) also emphasizes the identification of patients among individuals with obesity, prediabetes, or type 2 diabetes who have a higher likelihood of steatohepatitis and advanced fibrosis [4]. These recommendations are consistent with the American Gastroenterological Association (AGA) clinical care pathway and the American Diabetes Association (ADA) consensus report, both of which support multidisciplinary risk stratification and early intervention in metabolically high-risk populations [5,6].

The worldwide prevalence of steatotic liver disease has risen alongside the worldwide increase in obesity and type 2 diabetes. A systematic review and meta-analysis estimated that MASLD affects roughly one in three adults globally, with its prevalence increasing substantially over time [7]. The burden is particularly high among individuals with obesity or type 2 diabetes, who are also more likely to develop MASH and clinically significant fibrosis [2-7]​​​. Although simple steatosis may remain stable in some individuals, MASH represents a progressive inflammatory phenotype characterized histologically by hepatic steatosis, hepatocellular ballooning, and lobular inflammation, with variable degrees of fibrosis [1-3].

The clinical importance of MASH is primarily determined by the risk of progression to bridging fibrosis, cirrhosis, portal hypertension, hepatic decompensation, hepatocellular carcinoma, the need for liver transplantation, and death attributable to liver disease [2,3,8]. Among the histological components of the disease, the fibrosis stage is the most important determinant of liver-related illness and death. A large systematic review and meta-analysis demonstrated a stepwise increase in all-cause mortality and liver-related events with increasing fibrosis stage [8]. Consequently, resolution of MASH without progression of fibrosis and improvement in fibrosis without aggravation of MASH have become major histological efficacy endpoints in randomized clinical trials and regulatory drug-development programs.

The mechanisms underlying MASLD and MASH are multifactorial and reflect the interplay of excess adiposity, adipose tissue dysfunction, systemic insulin resistance, increased hepatic influx of free fatty acids, enhanced de novo lipogenesis, and impaired lipid oxidation, mitochondrial dysfunction, oxidative stress, endoplasmic reticulum stress, lipotoxicity, inflammatory signaling, and hepatic stellate cell activation [9]. Insulin resistance promotes adipose tissue lipolysis and increases the hepatic influx of free fatty acids, while hyperinsulinemia and excess substrate availability stimulate hepatic lipid synthesis. When the capacity to safely store or export lipids is exceeded, toxic lipid intermediates contribute to hepatocellular injury, ballooning, inflammation, and activation of fibrogenic pathways [9]. These mechanisms provide a strong rationale for therapeutic strategies capable of simultaneously reducing body weight, improving insulin sensitivity, modifying adipose tissue function, decreasing hepatic fat accumulation, and limiting progressive liver injury.

Lifestyle modification, dietary intervention, physical activity, and sustained weight reduction remain fundamental components of MASLD management according to current guidelines [2-5]. Histological improvement is closely related to the magnitude of weight loss. In a prospective lifestyle intervention study, greater weight reduction was associated with higher rates of steatohepatitis resolution and fibrosis regression, with the most substantial histological benefits observed among participants who achieved at least 10% total body weight loss [10]​​​​​​​. However, achieving and maintaining this degree of weight reduction is difficult in routine clinical practice, particularly among patients with severe obesity, diabetes, or multiple cardiometabolic comorbidities.

Pharmacological treatment of MASH has consequently become an important area of clinical investigation. Resmetirom, a liver-targeted selective agonist of thyroid hormone receptor beta, significantly improved both MASH resolution and liver fibrosis in a randomized phase 3 study involving patients with noncirrhotic MASH and moderate-to-advanced fibrosis [11]​​​​​​​. Its development represented a major milestone in MASH treatment. Nevertheless, MASLD remains a heterogeneous systemic metabolic disease, and considerable interest persists in therapies that may address both hepatic injury and the underlying cardiometabolic drivers of progression of the condition.

Glucagon-like peptide-1 (GLP-1) receptor agonists have become especially promising therapeutic option candidates because of their effects on glucose-dependent insulin secretion, glucagon regulation, appetite, gastric emptying, body weight, and insulin sensitivity [12]​​​​​​​. Early proof-of-concept trials demonstrated that selective GLP-1 receptor agonism could improve steatohepatitis activity. In the LEAN trial, liraglutide was associated with a higher frequency of NASH resolution and a lower rate of fibrosis progression than placebo [13]​​​​​​​. In a subsequent phase 2 trial, semaglutide significantly increased NASH resolution without worsening of fibrosis, although it did not produce a statistically significant improvement in fibrosis stage compared with placebo [14]​​​​​​​. These findings supported further evaluation of incretin-based therapies while also suggesting that greater metabolic efficacy or complementary receptor activation might be required to consistently affect fibrosis.

Dual and triple GLP-1-based polyagonists were designed to activate complementary hormonal pathways and potentially produce broader metabolic and hepatic effects than selective GLP-1 receptor agonists. Tirzepatide combined activation of the glucose-dependent insulinotropic polypeptide (GIP) and GLP-1 receptors. Survodutide, pemvidutide, cotadutide, and efinopegdutide combine GLP-1 and glucagon receptor agonism, although their relative receptor potency and pharmacological profiles differ. Retatrutide simultaneously activates the GIP, GLP-1, and glucagon receptors. In addition to the anorectic and glucose-lowering effects associated with incretin signaling, glucagon receptor agonism may enhance energy expenditure, hepatic lipid oxidation, and mobilization of intrahepatic triglyceride stores. The balance among GLP-1, GIP, and glucagon receptor activity may therefore influence body weight reduction, glycemic control, liver fat reduction, tolerability, and histological response.

Recent randomized controlled trials (RCTs) have generated encouraging evidence for GLP-1-based dual and triple polyagonists in MASLD and MASH. Tirzepatide and survodutide have shown significant benefits in biopsy-confirmed MASH, including higher rates of MASH resolution or improvement without worsening of fibrosis, with additional effects on hepatic fat content and body weight [15,16]. Pemvidutide, retatrutide, efinopegdutide, and cotadutide have also demonstrated favorable imaging-based, metabolic, biochemical, or preliminary histological effects, including reductions in liver fat content, body weight, aminotransferases, and markers of hepatic inflammation, although trial populations, comparators, endpoints, and histological assessment varied across studies [17-21]. Collectively, these findings suggest that dual and triple receptor agonism may reduce hepatic steatosis, improve metabolic dysfunction, promote weight loss, and, for selected agents, improve histological disease activity.

However, interpretation of the available evidence remains challenging. Trials differ in receptor targets, pharmacological potency, study population, diabetes status, baseline body mass index, fibrosis severity, drug dose, comparator, follow-up duration, and outcome definitions. Histological outcomes are available for only a subset of agents, whereas other trials have relied primarily on magnetic resonance imaging-proton density fat fraction (MRI-PDFF), serum aminotransferases, noninvasive fibrosis markers, or changes in body weight. MRI-PDFF is a sensitive and reproducible method for quantifying liver fat, but reduction in liver fat should not be considered equivalent to MASH resolution or fibrosis regression. Furthermore, the potential efficacy of polyagonists must be balanced against adverse effects, particularly nausea, vomiting, diarrhea, constipation, reduced appetite, and adverse events leading to treatment discontinuation. Gastrointestinal intolerance appears to be dose-dependent and may influence treatment adherence and the applicability of trial findings to routine clinical practice [15-21]​​​​​​​.

Previous evidence syntheses have evaluated selective GLP-1 receptor agonists or incretin-based therapies more broadly, but the rapidly expanding evidence for dual and triple polyagonists warrants a focused analysis [22]​​​​​​​. Pooling the available randomized evidence may improve statistical precision, clarify the magnitude and consistency of hepatic and metabolic benefits, and provide a more comprehensive assessment of safety. Nonetheless, differences between histological and imaging-based populations require careful outcome-specific analyses rather than treating all measures of hepatic response as interchangeable.

Accordingly, this systematic review and meta-analysis aimed to examine the efficacy and safety of dual and triple GLP-1-based polyagonists in adults with MASLD or MASH. Efficacy endpoints comprised MASH resolution or histological improvement without fibrosis progression, improvement in fibrosis without worsening of MASH, relative changes in liver fat content assessed by MRI-PDFF, achievement of a relative liver fat reduction of at least 30%, and percentage change in body weight. Safety endpoints included overall adverse events, gastrointestinal events such as nausea, diarrhea, vomiting, and constipation, serious adverse events, and adverse events resulting in treatment discontinuation.

## Review

Methods

Study Design and Search Strategy

A systematic literature search was conducted and reported in accordance with the Preferred Reporting Items for Systematic Reviews and Meta-Analyses (PRISMA 2020) guidelines [23]. The protocol was prospectively recorded in the International Prospective Register of Systematic Reviews (PROSPERO; CRD420261418862).

A systematic and electronic search of the literature was performed in PubMed and the Cochrane Central Register of Controlled Trials (CENTRAL) through the Cochrane Library, ScienceDirect, and Google Scholar from database inception through May 19, 2026. Database-specific search strategies are provided in Appendix 1 to improve transparency and reproducibility. The PubMed strategy was modified for CENTRAL by removing PubMed-specific field tags and Medical Subject Headings while retaining the principal disease, intervention, and study-design concepts. Google Scholar was used as a supplementary search engine to identify potentially eligible studies and records that might not have been retrieved through conventional bibliographic database searches. Because Google Scholar retrieves a large and heterogeneous number of results and does not provide the same advanced filtering and reproducibility features as conventional databases, the first 100 results ranked by relevance were screened. This limit was established as a pragmatic approach to maintain the feasibility and transparency of the supplementary search. The search results were sorted by relevance, and the first 100 results were screened using their titles, abstracts, and other available record information to assess potential eligibility. ScienceDirect was searched as a supplementary full-text and publisher-platform source. Because the platform limits the number of Boolean operators that can be used within a single search, separate searches were performed for each intervention using the following structure: (drug name) AND (MASLD OR MASH OR NAFLD OR NASH OR "fatty liver" OR steatohepatitis). The search was repeated using tirzepatide, survodutide, pemvidutide, efinopegdutide, cotadutide, and retatrutide as the intervention term. Results were restricted to research articles and screened by title and abstract to identify potentially eligible studies.

No language restrictions were applied. Embase was not available through the investigators’ institutional resources. To reduce the possibility of missing eligible trials, the bibliographies of the included studies and relevant systematic and narrative reviews were manually searched. Records retrieved from all sources were combined, and duplicate records were removed before title and abstract screening.

Study Selection

Study selection was carried out independently by two reviewers. Discrepancies were resolved through discussion and consensus, with input from a third reviewer when needed.

The electronic database searches yielded 652 records. After 16 duplicates were removed, 636 records proceeded to title and abstract screening, and 605 were excluded.

A total of 31 reports were sought for retrieval, of which two were unavailable for retrieval, leaving 29 full-text reports for eligibility evaluation. After reviewing the full texts, 23 reports were excluded: one because of an ineligible population, two because of an ineligible intervention, three because of an ineligible comparator, and 17 because they did not report eligible outcomes or provide sufficient outcome data for extraction. Ultimately, six RCTs fulfilled the prespecified eligibility criteria and were incorporated into both the qualitative and quantitative analyses.

Eligibility Criteria

RCTs that enrolled individuals 18 years of age or older with MASLD, MASH, NAFLD, or NASH were eligible for inclusion. Studies were required to evaluate a dual or triple GLP-1-based polyagonist and compare it with placebo, standard care, or an active comparator. Eligible interventions included tirzepatide, survodutide, pemvidutide, efinopegdutide, cotadutide, and retatrutide. Studies qualified for inclusion if they provided data on at least one relevant histological, liver imaging, metabolic, or safety outcome. For inclusion in a specific quantitative synthesis, studies were required to provide sufficient extractable data to calculate the corresponding effect estimate and measure of variance.

We excluded non-randomized studies, observational studies, reviews, editorials, letters, case reports, preclinical animal research, and in vitro investigations. Studies were also excluded if they did not enroll patients with MASLD, MASH, NAFLD, or NASH; did not evaluate an eligible dual or triple GLP-1-based polyagonist as the intervention of interest; or did not report an outcome relevant to this review. Trials evaluating GLP-1 receptor monoagonists, including semaglutide, liraglutide, dulaglutide, or exenatide, as the intervention of interest were excluded; however, such agents were permitted as active comparators. Studies that otherwise met the eligibility criteria but did not provide sufficient data for effect-size calculation were retained for qualitative assessment when applicable but were excluded from the corresponding quantitative synthesis.

Data Extraction

Data were extracted independently by two reviewers from all eligible studies using a predefined standardized form. The information collected included the first author, publication year, trial phase, and study design, study population, diagnostic criteria for MASLD, MASH, NAFLD, or NASH, total sample size, number of participants assigned to each treatment group, intervention, dose, route of administration, treatment duration, comparator, and duration of follow-up.

The outcomes of interest were defined before data extraction. Outcomes were included in the quantitative synthesis when they were reported by a sufficient number of studies and were considered clinically and methodologically comparable. Histological outcomes included MASH resolution or improvement without worsening of fibrosis, as defined in the original trials, and a reduction of one or more fibrosis stages without progression of MASH. Imaging outcomes comprised the relative percentage difference relative to baseline in liver fat content measured by magnetic resonance imaging-proton density fat fraction (MRI-PDFF) and the proportion of participants achieving a relative liver fat reduction of at least 30%. The metabolic outcome was the percentage change from baseline in body weight.

Safety outcomes included any adverse event, nausea, diarrhea, vomiting, constipation, serious adverse events, and adverse events leading to treatment discontinuation. Outcomes reported by an insufficient number of studies or defined or presented in a manner that precluded clinically meaningful pooling were summarized narratively, when applicable, and were not included in the quantitative synthesis.

For dichotomous outcomes, we extracted the count of participants experiencing each event and the total evaluated in each study group. For continuous outcomes, we recorded the mean change from baseline, the corresponding standard deviation or standard error, and the number of participants included in the analysis. When standard deviations were unavailable, they were calculated from reported standard errors, confidence intervals, or other summary statistics following methods described in the Cochrane Handbook for Systematic Reviews of Interventions [24].

Whenever possible, data from the intention-to-treat or modified intention-to-treat population were used for efficacy outcomes, whereas data from the safety population were used for adverse-event outcomes. When multiple follow-up assessments were available, data obtained at the end of the randomized treatment period were extracted.

For trials with multiple eligible intervention-dose groups and a shared comparator group, data were extracted separately for each eligible dose group. To avoid double counting participants in the shared comparator group, the comparator group was divided evenly across the relevant pairwise comparisons in line with the procedures outlined in the Cochrane Handbook.

Discrepancies between the two reviewers were resolved by discussion until consensus was reached. When agreement remained unresolved, a third reviewer provided the final decision.

Risk of Bias Assessment

We evaluated the risk of bias of the included studies using the Cochrane Risk of Bias 2 (RoB 2) tool [25]. The assessment included five areas of evaluation: bias arising from the randomization process, deviations from intended interventions, missing outcome data, measurement of the outcome, and selection of the reported result.

Statistical Analysis

All meta-analyses were performed using Review Manager software (RevMan version 5.4; The Cochrane Collaboration, Copenhagen, Denmark). Dual and triple GLP-1-based polyagonists were compared with placebo across histological, imaging, metabolic, and safety outcomes.

Dichotomous outcomes were analyzed using risk ratios (RRs) with 95% confidence intervals (CIs) and the Mantel-Haenszel method. These outcomes included MASH resolution or improvement without worsening of fibrosis, as defined in the original trials; improvement of at least one fibrosis stage without worsening of MASH; and an imaging response defined as a relative reduction of at least 30% in liver fat content measured by magnetic resonance imaging-proton density fat fraction (MRI-PDFF). Safety outcomes analyzed as dichotomous variables included any adverse event, nausea, diarrhea, vomiting, constipation, serious adverse events, and adverse events leading to treatment discontinuation.

Continuous outcomes were analyzed using mean differences (MDs) with 95% CIs and the inverse-variance method because the included studies reported these outcomes using comparable measurement scales. Continuous outcomes included the relative percentage change from baseline in liver fat content measured by MRI-PDFF and the percentage change from baseline in body weight.

Given the anticipated clinical and methodological variability across the included trials, such as differences in the intervention, dosage, treatment duration, and follow-up period, baseline liver disease severity, and outcome definitions and assessment methods, random-effects models were used for all pooled analyses. Statistical heterogeneity was assessed using Cochran’s Chi-square test and quantified using the I² statistic. A p-value below 0.10 for the Chi-square test was interpreted as evidence of statistical heterogeneity, and I² values greater than 50% were considered to represent substantial heterogeneity.

For multi-arm trials contributing more than one eligible intervention-dose group to the same meta-analysis, the shared placebo group was divided evenly across the relevant pairwise comparisons to avoid double counting, following the procedures recommended in the Cochrane Handbook for Systematic Reviews of Interventions. Sensitivity analyses were planned to assess the stability of the pooled estimates but were not conducted because only a small number of studies contributed to each meta-analysis.

Publication bias was not evaluated with funnel plots because less than 10 studies contributed to each pooled outcome, limiting the reliability of visual asymmetry assessment. All overall-effect tests were two-sided, and p values below 0.05 were considered statistically significant.

Results

Search Results and Study Selection

The literature search identified a total of 652 records through database searching, including 17 records from PubMed, 151 from CENTRAL, 384 from ScienceDirect, and 100 from Google Scholar. After the removal of 16 duplicate records, 636 records were screened based on title and abstract. Of these, 605 records were excluded because they were not relevant to the review question or did not meet the predefined inclusion criteria.

**Figure 1:**
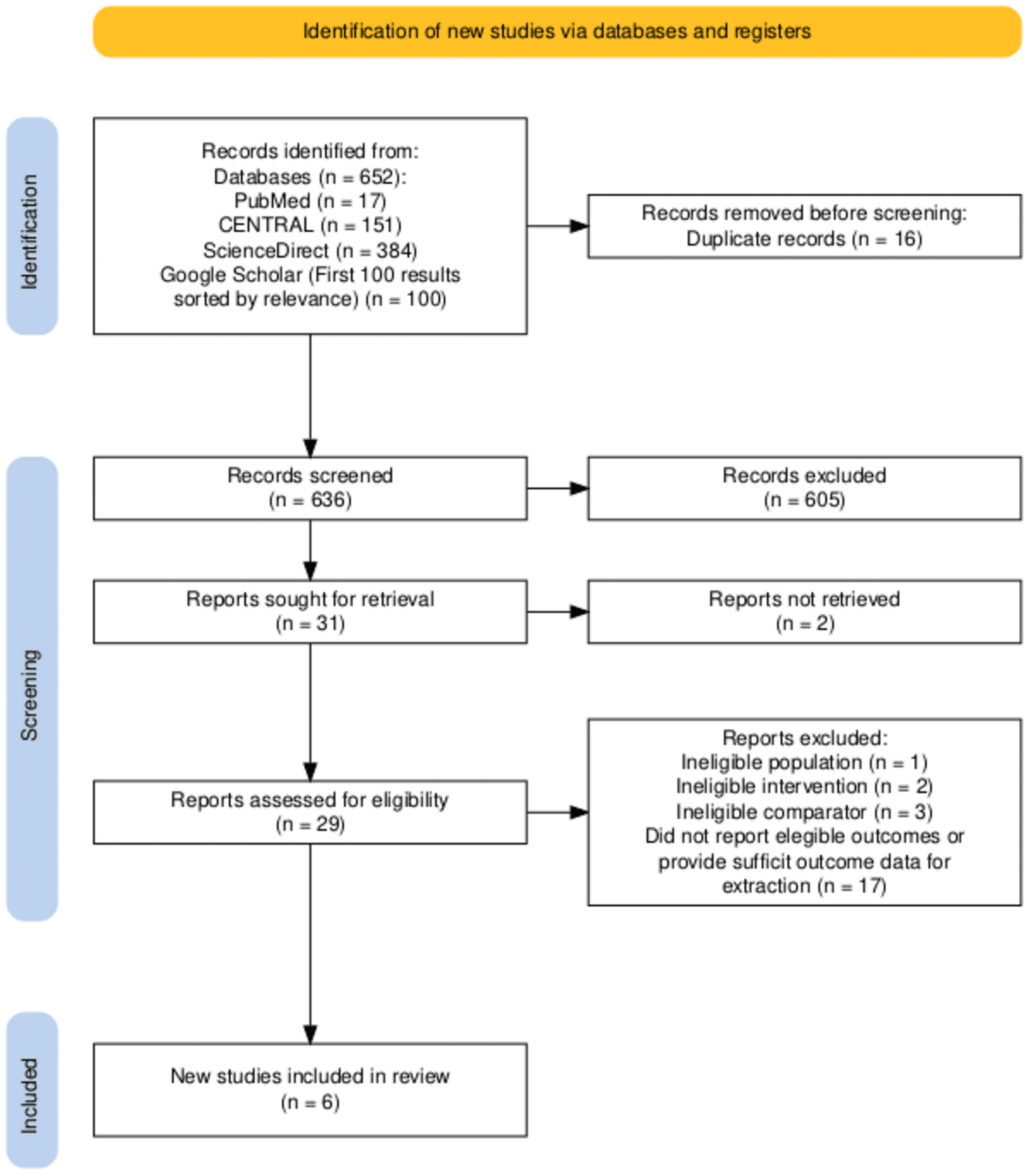
PRISMA 2020 flow diagram of study selection. Flow diagram constructed according to PRISMA 2020 guidelines [23]. PRISMA: Preferred Reporting Items for Systematic Reviews and Meta-Analyses.

A total of 31 full-text reports were requested for retrieval. Of these, two could not be retrieved, leaving 29 full-text articles for eligibility assessment. After full-text review, 23 reports were excluded: one because of an ineligible population, two because of an ineligible intervention, three because of an ineligible comparator, and 17 because they did not report eligible outcomes or provide sufficient data for extraction. Ultimately, six RCTs were included in the qualitative and quantitative synthesis (Figure 1).

Study Characteristics

The final analysis included six randomized, double-blind, placebo-controlled trials evaluating dual or triple GLP-1-based polyagonists in adults with MASLD or MASH [15-18,20,21]. A total of 961 participants were randomized across the included trials. The studies were conducted in multicenter or multinational settings and evaluated tirzepatide, survodutide, pemvidutide, retatrutide, and cotadutide. Treatment duration ranged from 12 to 52 weeks.

The included trials differed in study phase, diagnostic criteria, baseline liver disease severity, intervention, dose, treatment duration, and outcome assessment. Four trials enrolled participants with biopsy-confirmed MASH and fibrosis, whereas two enrolled participants with MASLD defined primarily by elevated liver fat content quantified by magnetic resonance imaging-proton density fat fraction (MRI-PDFF). Three trials reported histological efficacy outcomes, while the remaining studies primarily evaluated changes in liver fat content and other noninvasive hepatic and metabolic outcomes.

Three studies contributed data to the analyses of MASH resolution or improvement without worsening of fibrosis and improvement of at least one fibrosis stage without worsening of MASH. Three studies reported relative percentage changes in liver fat content, and three reported the proportion of participants achieving a relative reduction of at least 30% in liver fat content measured by MRI-PDFF. Two studies provided sufficiently comparable data for the percentage change from baseline in body weight. Depending on the outcome, between three and five studies contributed data to the pooled safety analyses.

Individual trial sample sizes ranged from 74 to 293 participants. Differences in study populations, intervention mechanisms, dose regimens, treatment duration, baseline liver disease severity, and outcome definitions should be considered when interpreting the pooled estimates. The key features of the included RCTs are summarized in Table 1.

**Table 1:** Characteristics of the randomized controlled trials evaluating dual and triple GLP-1–based polyagonists in MASLD or MASH This table summarizes the main characteristics of the six randomized, double-blind, placebo-controlled trials included in the qualitative and quantitative syntheses, including study setting, population, sample size, intervention and comparator groups, treatment duration, primary outcomes, and key findings. Sample sizes are presented as intervention/placebo groups, as reported in the original studies [15-18,20,21]. BMI: body mass index; F: fibrosis stage; GLP-1: glucagon-like peptide-1; MASH: metabolic dysfunction-associated steatohepatitis; MASLD: metabolic dysfunction-associated steatotic liver disease; MRI-PDFF: magnetic resonance imaging–proton density fat fraction.

Study	Year	Setting	Population	Sample size, intervention/placebo	Intervention	Duration	Primary outcome	Key findings
Harrison et al. [17]	2025	Multicenter	MASLD; BMI ≥28 kg/m²; liver fat content ≥10% by MRI-PDFF	70/24	Pemvidutide 1.2, 1.8, or 2.4 mg once weekly vs. placebo	12 weeks	Relative change in liver fat content	Significant reduction in liver fat content and body weight
Loomba et al. [15]	2024	International, multicenter	Biopsy-confirmed MASH with F2–F3 fibrosis	142/48	Tirzepatide 5, 10, or 15 mg once weekly vs. placebo	52 weeks	MASH resolution without worsening of fibrosis	Significant improvement in MASH resolution and fibrosis outcomes
Noureddin et al. [18]	2025	United States and Australia, multicenter	Biopsy-confirmed MASH with F2–F3 fibrosis	126/86	Pemvidutide 1.2 or 1.8 mg once weekly vs. placebo	24 weeks	MASH resolution and fibrosis improvement	Significant improvement in MASH resolution; no significant fibrosis benefit at week 24
Sanyal et al. [16]	2024	International, multicenter	Biopsy-confirmed MASH with F1–F3 fibrosis	219/74	Survodutide 2.4, 4.8, or 6.0 mg once weekly vs. placebo	48 weeks	MASH improvement without worsening of fibrosis	Significant histological improvement and liver fat reduction
Sanyal et al. [20]	2024	MASLD substudy of an obesity trial	Obesity with liver fat content ≥10% by MRI-PDFF	79/19	Retatrutide 1, 4, 8, or 12 mg once weekly vs. placebo	48 weeks	Relative change in liver fat content at week 24	Marked dose-dependent reductions in liver fat and body weight
Shankar et al. [21]	2024	United States and Puerto Rico, multicenter	Obesity with biopsy-confirmed noncirrhotic MASH and F1–F3 fibrosis	50/24	Cotadutide 300 or 600 μg once daily vs. placebo	19 weeks	Safety and hepatic fat fraction	Reduction in hepatic fat and liver injury markers; gastrointestinal adverse events were common

Quality Assessment

The risk of bias of the included RCTs was independently assessed by two reviewers using the Cochrane Risk of Bias 2 (RoB 2) tool. Discrepancies were resolved by discussion until consensus was reached, with involvement of a third reviewer when necessary. The evaluation included five areas of assessment: bias related to the randomization process, deviations from the intended interventions, missing outcome data, outcome measurement, and selective reporting of results. Overall risk-of-bias judgments were categorized as low risk, some concerns, or high risk according to the RoB 2 criteria.

Overall, one study was rated with a low overall risk of bias, whereas five raised some concerns. None of the included trials was judged to have a high overall risk of bias. Across all studies, the domains related to randomization, deviations from intended interventions, and outcome measurement were rated as low risk.

The most frequent concerns were identified in Domain 3 because several trials had incomplete post-treatment biopsy or MRI-PDFF assessments, used modified intention-to-treat populations, or required the availability of post-baseline measurements for inclusion in efficacy analyses. Although prespecified imputation methods, non-responder assumptions, or longitudinal statistical models were generally applied, the possibility that missing outcome data were related to treatment discontinuation, tolerability, or the true outcome could not be completely excluded.

**Figure 2:**
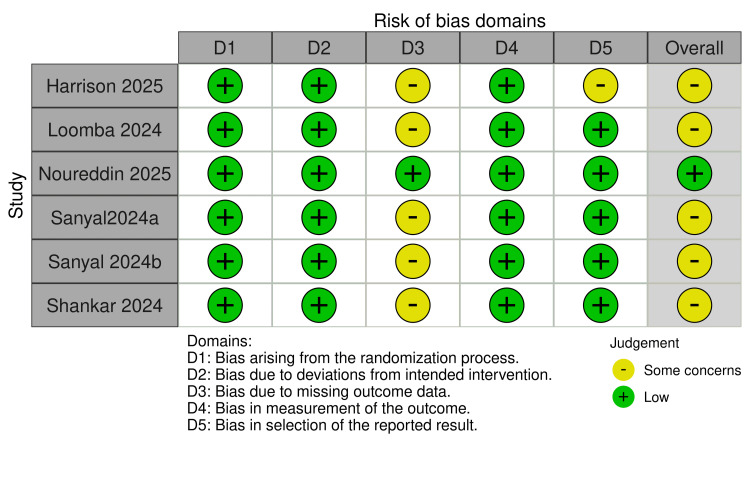
Risk-of-bias assessment of included studies using the Cochrane Risk of Bias 2 (RoB 2) tool. The individual risk-of-bias judgments for each included study. This figure (traffic light plot) summarizes the risk of bias (RoB) assessments in each domain of the Cochrane RoB 2 tool across all six included studies [15-18,20,21]. RoB 2 tool: risk of bias 2 tool; D1: bias arising from the randomization process; D2: bias due to deviations from intended interventions; D3: bias due to missing outcome data; D4: bias in the measurement of the outcome; D5: bias in the selection of the reported result.

**Figure 3:**
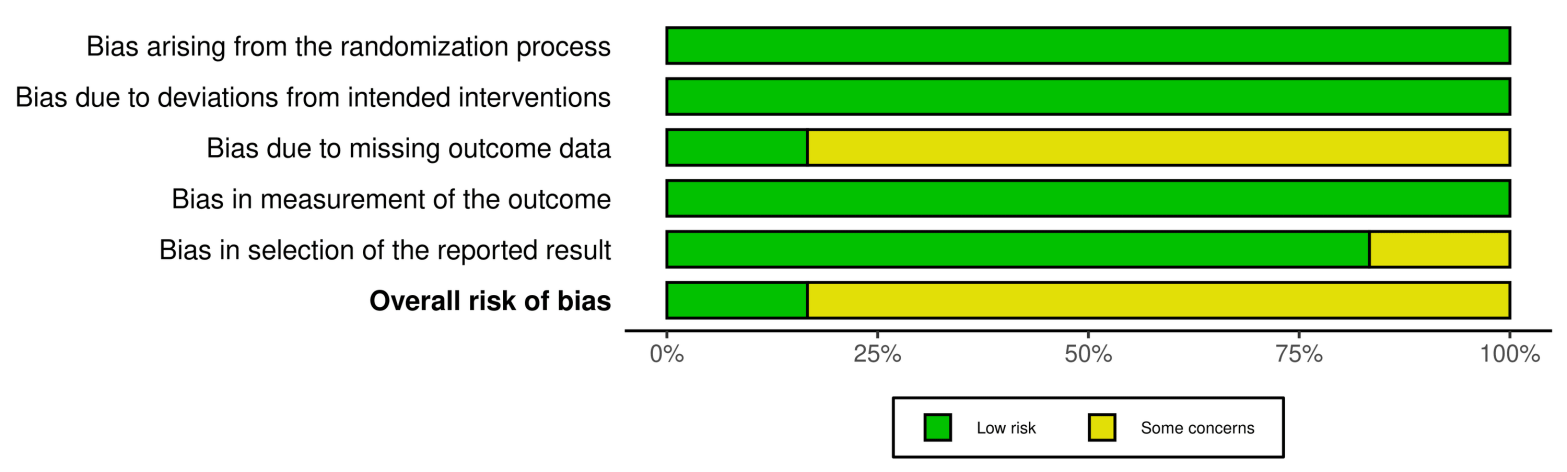
Summary of the risk-of-bias assessment of the included randomized controlled trials. The figure presents the proportion of included studies rated as having a low risk of bias or some concerns across the five domains of the Cochrane Risk of Bias 2 tool and for the overall risk-of-bias judgment. Green indicates low risk of bias, and yellow indicates some concerns. No study was judged to be at high risk of bias. Six randomized controlled trials were included [15-18,20,21].

Some concerns regarding Domain 5 were identified in one study because selected categorical responder analyses were reported as post hoc analyses. The remaining trials generally reported outcomes that were consistent with their registered protocols, prespecified endpoints, or statistical analysis plans. The summary risk-of-bias graph and the individual study-level judgments are presented in Figures 2, 3.

MASH Resolution or Improvement Without Worsening of Fibrosis

The meta-analysis included three RCTs comprising 695 participants of MASH resolution or histological improvement without worsening of fibrosis [15,16,18]. Pooled analysis using a random-effects model showed that dual or triple GLP-1-based polyagonists significantly increased the likelihood of achieving MASH resolution or improvement without worsening of fibrosis compared with placebo: RR = 3.32, 95% CI 2.28-4.84; I² = 20%; p < 0.00001.

**Figure 4:**
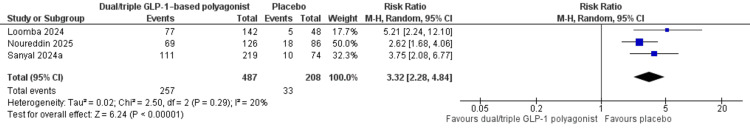
Forest plot of MASH resolution or improvement without worsening of fibrosis comparing dual or triple GLP-1–based polyagonists with placebo. Forest plot showing the effect of dual or triple GLP-1-based polyagonists versus placebo on MASH resolution or histological improvement without worsening of fibrosis, using risk ratios and a random-effects model. Risk ratios greater than 1 favor dual or triple GLP-1-based polyagonists, whereas risk ratios less than 1 favor placebo. Three randomized controlled trials were included [15,16,18]. CI: confidence interval; df: degrees of freedom; M-H: Mantel-Haenszel; MASH: metabolic dysfunction-associated steatohepatitis; RR: risk ratio.

Heterogeneity was low, indicating generally consistent treatment effects across the included studies. Overall, dual or triple GLP-1-based polyagonists were associated with a significantly greater likelihood of histological MASH improvement without worsening of fibrosis compared with placebo (Figure 4).

Fibrosis Improvement Without Worsening of MASH

The meta-analysis included three RCTs comprising 695 participants of fibrosis improvement without worsening of MASH [15,16,18]. Pooled analysis using a random-effects model showed that dual or triple GLP-1-based polyagonists significantly increased the likelihood of achieving at least one-stage fibrosis improvement without worsening of MASH compared with placebo: RR = 1.49, 95% CI 1.15-1.94; I² = 0%; p = 0.002.

**Figure 5:**
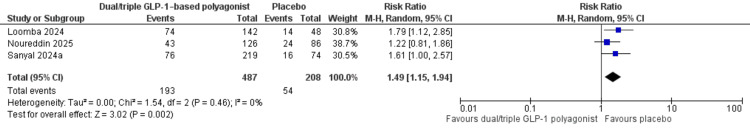
Forest plot of fibrosis improvement without worsening of MASH comparing dual or triple GLP-1–based polyagonists with placebo. Forest plot showing the effect of dual or triple GLP-1-based polyagonists versus placebo on improvement of at least one fibrosis stage without worsening of MASH, using risk ratios and a random-effects model. Risk ratios greater than 1 favor dual or triple GLP-1–based polyagonists, whereas risk ratios less than 1 favor placebo. Three randomized controlled trials were included [15,16,18]. CI: confidence interval; df: degrees of freedom; M-H: Mantel-Haenszel; MASH: metabolic dysfunction-associated steatohepatitis; RR: risk ratio.

No statistical heterogeneity was observed, indicating a highly consistent treatment effect across the included studies. Overall, dual or triple GLP-1-based polyagonists were associated with a significantly higher probability of fibrosis improvement without worsening of MASH compared with placebo (Figure 5).

Change in Liver Fat Content: Relative Percentage Change

The meta-analysis comprised three RCTs with a total of 485 participants of relative percentage change in liver fat content [15,17,18]. Pooled analysis using a random-effects model showed that dual or triple GLP-1-based polyagonists produced a markedly higher relative reduction in liver fat content compared with placebo: MD = −44.60 percentage points, 95% CI −51.17 to −38.04; I² = 0%; p < 0.00001.

**Figure 6:**
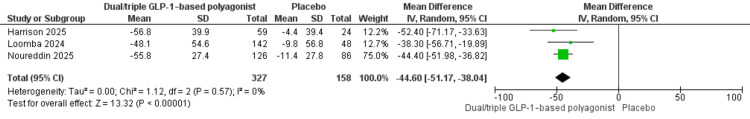
Forest plot of relative percentage change in liver fat content comparing dual or triple GLP-1-based polyagonists with placebo. Forest plot showing the effect of dual or triple GLP-1–based polyagonists versus placebo on the relative percentage change from baseline in liver fat content, using mean differences and a random-effects model. Negative mean differences favor dual or triple GLP-1-based polyagonists, whereas positive mean differences favor placebo. Three randomized controlled trials were included [15,17,18]. CI: confidence interval; df: degrees of freedom; IV: inverse variance; MD: mean difference.

No statistical heterogeneity was observed, indicating a highly consistent treatment effect across the included studies. Overall, dual or triple GLP-1-based polyagonists were associated with a substantial reduction in liver fat content relative to placebo (Figure 6).

≥30% Relative Reduction in Liver Fat Content by MRI-PDFF

The meta-analysis included three RCTs encompassing 570 participants achieving at least a 30% relative reduction in liver fat content measured by MRI-PDFF [16-18]. Pooled analysis using a random-effects model showed that dual or triple GLP-1-based polyagonists produced a significantly greater probability of achieving this imaging response than placebo: RR = 4.71, 95% CI 3.04-7.30; I² = 22%; p < 0.00001.

**Figure 7:**
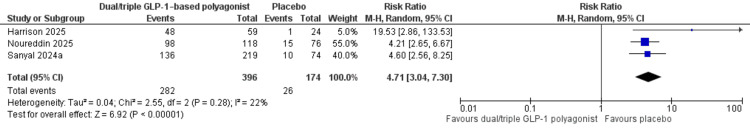
Forest plot of ≥30% relative reduction in liver fat content by MRI-PDFF comparing dual or triple GLP-1-based polyagonists with placebo. Forest plot showing the effect of dual or triple GLP-1-based polyagonists versus placebo on the proportion of participants achieving at least a 30% relative reduction from baseline in liver fat content measured by MRI-PDFF, using risk ratios and a random-effects model. Risk ratios greater than 1 favor dual or triple GLP-1-based polyagonists, whereas risk ratios less than 1 favor placebo. Three randomized controlled trials were included [16-18]. CI: confidence interval; df: degrees of freedom; M-H: Mantel-Haenszel; MRI-PDFF: magnetic resonance imaging–proton density fat fraction; RR: risk ratio.

Heterogeneity was low, indicating generally consistent treatment effects across the included studies. Overall, participants receiving dual or triple GLP-1-based polyagonists were substantially more likely to achieve a clinically meaningful reduction in liver fat content than those receiving placebo (Figure 7).

Change in Body Weight: Percentage Change

The meta-analysis comprised two RCTs with a total of 310 participants of percentage change in body weight [18,20]. Pooled analysis using a random-effects model showed that dual or triple GLP-1-based polyagonists produced a markedly larger decrease in body weight compared with placebo: MD = −9.14 percentage points, 95% CI −17.38 to −0.91; I² = 98%; p = 0.03.

**Figure 8:**
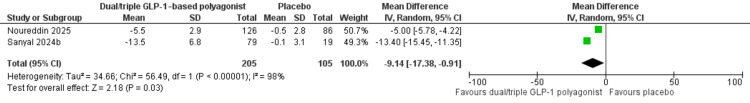
Forest plot of percentage change in body weight comparing dual or triple GLP-1-based polyagonists with placebo. Forest plot showing the effect of dual or triple GLP-1–based polyagonists versus placebo on the percentage change from baseline in body weight, using mean differences and a random-effects model. Negative mean differences favor dual or triple GLP-1-based polyagonists, whereas positive mean differences favor placebo. Two randomized controlled trials were included [18,20]. CI: confidence interval; df: degrees of freedom; IV: inverse variance; MD: mean difference; SD: standard deviation.

Heterogeneity was considerable, indicating substantial variability in the magnitude of weight reduction across the included studies. This heterogeneity may reflect differences in the polyagonist evaluated, treatment duration, dose, and baseline population characteristics. Although the pooled estimate favored dual or triple GLP-1-based polyagonists, the result should be interpreted cautiously because only two studies contributed to the analysis and statistical heterogeneity was very high (Figure 8).

Any Adverse Event

Four RCTs involving 770 participants contributed data to the meta-analysis of any adverse event [15,16,18,21]. Under a random-effects model, the pooled estimate indicated no statistically significant difference in the risk of experiencing any adverse event between dual or triple GLP-1-based polyagonists and placebo: RR = 1.17, 95% CI 0.98-1.39; I² = 79%; p = 0.08.

**Figure 9:**
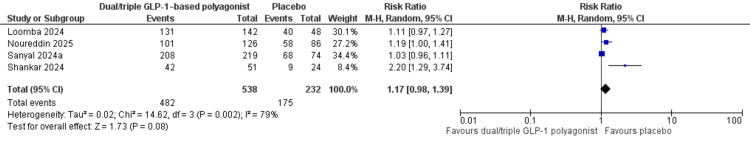
Forest plot of any adverse event comparing dual or triple GLP-1-based polyagonists with placebo. Forest plot showing the effect of dual or triple GLP-1-based polyagonists versus placebo on the occurrence of any adverse event, using risk ratios and a random-effects model. Risk ratios greater than 1 favor placebo, indicating a higher risk of adverse events with dual or triple GLP-1-based polyagonists, whereas risk ratios less than 1 favor the polyagonist group. Four randomized controlled trials were included [15,16,18,21]. CI: confidence interval; df: degrees of freedom; M-H: Mantel-Haenszel; RR: risk ratio.

Heterogeneity was substantial, indicating considerable variability in overall adverse-event rates across the included studies. This variability may reflect differences in the polyagonist evaluated, dose, treatment duration, adverse-event definitions, and study populations. Although the pooled estimate suggested a numerically higher risk with dual or triple GLP-1-based polyagonists, the confidence interval included the null effect, and the result was not statistically significant (Figure 9).

Nausea

Four RCTs comprising 674 participants were included in the meta-analysis of nausea [16-18,21]. Pooled analysis using a random-effects model showed that dual or triple GLP-1-based polyagonists were linked to a substantially increased risk of nausea compared with placebo: RR = 2.90, 95% CI 2.12-3.97; I² = 0%; p < 0.00001.

**Figure 10:**
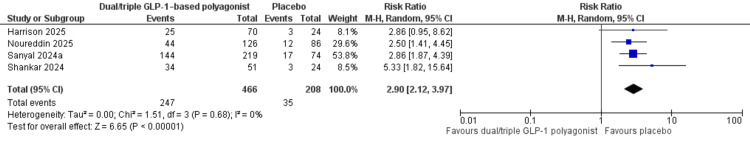
Forest plot of nausea comparing dual or triple GLP-1-based polyagonists with placebo. Forest plot showing the effect of dual or triple GLP-1-based polyagonists versus placebo on the occurrence of nausea, using risk ratios and a random-effects model. Risk ratios greater than 1 favor placebo, indicating a higher risk of nausea with dual or triple GLP-1-based polyagonists, whereas risk ratios less than 1 favor the polyagonist group. Four randomized controlled trials were included [16-18,21]. CI: confidence interval; df: degrees of freedom; M-H: Mantel-Haenszel; RR: risk ratio.

No statistical heterogeneity was observed, indicating a consistent increase in nausea across the included studies. Overall, nausea was significantly more frequent among participants receiving dual or triple GLP-1-based polyagonists than among those receiving placebo (Figure 10).

Diarrhea

Four RCTs involving 674 participants contributed data to the meta-analysis of diarrhea [16-18,21]. Pooled analysis using a random-effects model showed that dual or triple GLP-1-based polyagonists resulted in a significantly increased risk of diarrhea compared with placebo: RR = 1.90, 95% CI 1.24-2.89; I² = 13%; p = 0.003.

**Figure 11:**
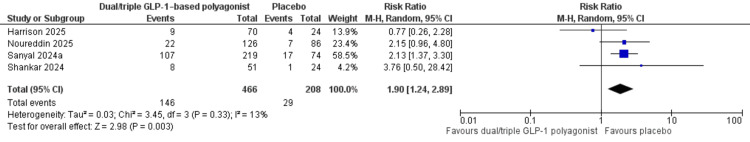
Forest plot of diarrhea comparing dual or triple GLP-1-based polyagonists with placebo. Forest plot showing the effect of dual or triple GLP-1-based polyagonists versus placebo on the occurrence of diarrhea, using risk ratios and a random-effects model. Risk ratios greater than 1 favor placebo, indicating a higher risk of diarrhea with dual or triple GLP-1-based polyagonists, whereas risk ratios less than 1 favor the polyagonist group. Four randomized controlled trials were included [16-18,21]. CI: confidence interval; df: degrees of freedom; M-H: Mantel-Haenszel; RR: risk ratio.

Heterogeneity was low, indicating generally consistent treatment effects across the included studies. Overall, diarrhea occurred significantly more frequently among participants receiving dual or triple GLP-1-based polyagonists than among those receiving placebo (Figure 11).

Vomiting

Three RCTs involving 462 participants contributed data to the meta-analysis of vomiting [16,17,21]. Pooled analysis using a random-effects model showed that dual or triple GLP-1-based polyagonists were associated with a significantly higher risk of vomiting compared with placebo: RR = 9.59, 95% CI 3.62-25.42; I² = 0%; p < 0.00001.

**Figure 12:**
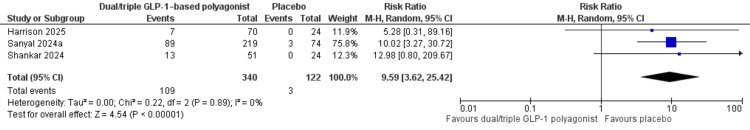
Forest plot of vomiting comparing dual or triple GLP-1-based polyagonists with placebo. Forest plot showing the effect of dual or triple GLP-1-based polyagonists versus placebo on the occurrence of vomiting, using risk ratios and a random-effects model. Risk ratios greater than 1 favor placebo, indicating a higher risk of vomiting with dual or triple GLP-1-based polyagonists, whereas risk ratios less than 1 favor the polyagonist group. Three randomized controlled trials were included [16,17,21]. CI: confidence interval; df: degrees of freedom; M-H: Mantel-Haenszel; RR: risk ratio.

No statistical heterogeneity was observed, indicating a consistent increase in vomiting across the included studies. Overall, vomiting was significantly more frequent among participants receiving dual or triple GLP-1-based polyagonists than among those receiving placebo (Figure 12).

Constipation

Three RCTs involving 599 participants contributed data to the meta-analysis of constipation [16-18]. Pooled analysis using a random-effects model showed no statistically significant difference in the risk of constipation between dual or triple GLP-1-based polyagonists and placebo: RR = 1.46, 95% CI 0.91-2.35; I² = 0%; p = 0.12.

**Figure 13:**
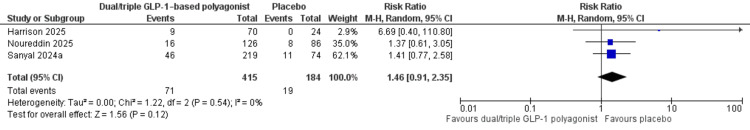
Forest plot of constipation comparing dual or triple GLP-1-based polyagonists with placebo. Forest plot showing the effect of dual or triple GLP-1-based polyagonists versus placebo on the occurrence of constipation, using risk ratios and a random-effects model. Risk ratios greater than 1 favor placebo, indicating a higher risk of constipation with dual or triple GLP-1-based polyagonists, whereas risk ratios less than 1 favor the polyagonist group. Three randomized controlled trials were included [16-18]. CI: confidence interval; df: degrees of freedom; M-H: Mantel-Haenszel; RR: risk ratio.

No statistical heterogeneity was observed, indicating consistent findings across the included studies. Although constipation occurred numerically more often among participants receiving dual or triple GLP-1-based polyagonists, the confidence interval included the null effect, and the difference was not statistically significant (Figure 13).

Serious Adverse Events

Five RCTs involving 864 participants contributed data to the meta-analysis of serious adverse events [15-18,21]. Pooled analysis using a random-effects model showed no statistically significant difference in the risk of serious adverse events between dual or triple GLP-1-based polyagonists and placebo: RR = 1.05, 95% CI 0.54-2.01; I² = 0%; p = 0.89.

**Figure 14:**
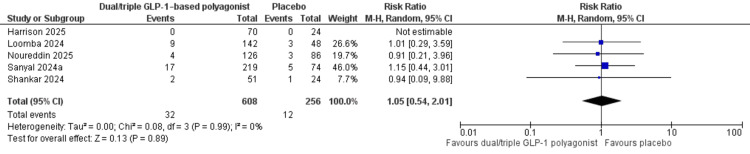
Forest plot of serious adverse events comparing dual or triple GLP-1-based polyagonists with placebo. Forest plot showing the effect of dual or triple GLP-1-based polyagonists versus placebo on the occurrence of serious adverse events, using risk ratios and a random-effects model. Risk ratios greater than 1 favor placebo, indicating a higher risk of serious adverse events with dual or triple GLP-1-based polyagonists, whereas risk ratios less than 1 favor the polyagonist group. Five randomized controlled trials were included [15-18,21]. CI: confidence interval; df: degrees of freedom; M-H: Mantel-Haenszel; RR: risk ratio.

No statistical heterogeneity was observed, indicating consistent findings across the included studies. Overall, dual or triple GLP-1-based polyagonists were not associated with a significantly increased risk of serious adverse events compared with placebo (Figure 14).

Adverse Events Leading to Treatment Discontinuation

Five RCTs involving 864 participants contributed data to the meta-analysis of adverse events leading to treatment discontinuation [15-18,21]. Pooled analysis using a random-effects model showed no statistically significant difference in the risk of treatment discontinuation due to adverse events between dual or triple GLP-1-based polyagonists and placebo: RR = 1.89, 95% CI 0.60-5.88; I² = 41%; p = 0.27.

**Figure 15:**
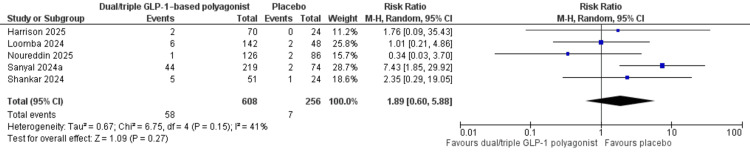
Forest plot of adverse events leading to treatment discontinuation comparing dual or triple GLP-1-based polyagonists with placebo. Forest plot showing the effect of dual or triple GLP-1-based polyagonists versus placebo on adverse events leading to treatment discontinuation, using risk ratios and a random-effects model. Risk ratios greater than 1 favor placebo, indicating a higher risk of treatment discontinuation with dual or triple GLP-1-based polyagonists, whereas risk ratios less than 1 favor the polyagonist group. Five randomized controlled trials were included [15-18,21]. CI: confidence interval; df: degrees of freedom; M-H: Mantel-Haenszel; RR: risk ratio.

Heterogeneity was moderate, suggesting some variability in discontinuation rates across the included studies. Although the pooled estimate numerically favored placebo, indicating a higher rate of discontinuation with dual or triple GLP-1-based polyagonists, the confidence interval was wide and included the null effect. Overall, dual or triple GLP-1-based polyagonists were not associated with a statistically significant increase in adverse events leading to treatment discontinuation compared with placebo (Figure 15).

Publication Bias

Because only six RCTs were included overall, and fewer than 10 studies contributed to each pooled outcome, formal evaluation of publication bias through funnel plots or statistical methods for funnel plot asymmetry was not considered reliable. Therefore, publication bias was not formally assessed.

Although no definitive conclusion regarding publication bias can be drawn, the small number of studies contributing to each outcome and the modest sample sizes of several trials warrant cautious interpretation of the pooled estimates.

Certainty of Evidence (Grading of Recommendations Assessment, Development, and Evaluation (GRADE))

We applied the GRADE methodology to determine the level of confidence in the principal efficacy and safety findings. Because the evidence originated from randomized trials, each outcome entered the assessment at a high-certainty level. The rating was subsequently reduced whenever concerns were found regarding methodological bias, variability across studies, limited applicability, uncertainty in the effect estimates, or possible publication bias. Priority was given to outcomes considered clinically relevant for therapeutic decision-making.

The certainty of evidence for MASH resolution or histological improvement without progression of fibrosis was considered moderate. The pooled result significantly favored dual or triple GLP-1-based polyagonists, with low between-study heterogeneity (I² = 20%). However, certainty was reduced by one level because of risk-of-bias concerns, mainly incomplete post-treatment biopsy assessments and the use of imputation or non-responder methods to address missing histological outcomes.

Evidence for fibrosis improvement without worsening of MASH was likewise rated as moderate certainty. The pooled effect favored dual or triple GLP-1-based polyagonists, heterogeneity was absent (I² = 0%), and the confidence interval did not cross the line of no effect. Even so, the evidence was downgraded by one level because of incomplete follow-up biopsy assessments and concerns about the methods used to manage missing outcome data.

The certainty of evidence for relative percentage change in liver fat content and achievement of at least a 30% relative reduction in liver fat content measured by MRI-PDFF was rated as moderate. Both outcomes demonstrated large, statistically significant treatment effects with low or no statistical heterogeneity. The evidence was downgraded by one level because some contributing trials used modified intention-to-treat populations or restricted efficacy analyses to participants with available post-baseline imaging assessments, resulting in concerns regarding missing outcome data.

For percentage change in body weight, the certainty of evidence was rated as low. Although the pooled estimate favored dual or triple GLP-1-based polyagonists, statistical heterogeneity was considerable (I² = 98%), indicating substantial variability in the magnitude of weight reduction across studies. The confidence interval was also wide and extended from a modest to a very large treatment effect. The evidence was therefore downgraded for serious inconsistency and imprecision.

Safety outcomes prioritized for the GRADE assessment were any adverse event, serious adverse events, and adverse events leading to treatment discontinuation. These outcomes were selected because they represent the overall burden of adverse events, clinically severe harm, and treatment-limiting tolerability, respectively. Individual gastrointestinal adverse events, including nausea, diarrhea, and vomiting, were evaluated as secondary safety outcomes but were not graded separately to avoid redundancy and to maintain focus on the safety outcomes considered most important for clinical decision-making.

The certainty of evidence for any adverse event was rated as low because of substantial statistical heterogeneity (I² = 79%) and imprecision. The confidence interval encompassed both little or no difference and a potentially clinically relevant increase in the overall incidence of adverse events.

The certainty of evidence for serious adverse events was rated as low. Although no statistical heterogeneity was observed, the number of events was small, and the confidence interval was wide, encompassing both a potentially important reduction and a clinically relevant increase in serious adverse events. The evidence was downgraded for risk-of-bias concerns and serious imprecision.

Evidence regarding adverse events leading to treatment discontinuation was also rated as low because of risk-of-bias concerns and serious imprecision. The pooled estimate was based on relatively few events, and its wide confidence interval encompassed little or no difference as well as a potentially substantial increase in treatment discontinuation.

Overall, the available evidence provides moderate certainty that dual and triple GLP-1-based polyagonists improve histological and imaging-based hepatic outcomes in adults with MASLD or MASH. However, certainty regarding body weight and safety outcomes remains low because of inconsistency, imprecision, and the limited amount of information provided by the available trials. A summary of the GRADE assessments is presented in Table 2.

**Table 2:** Summary of findings and certainty of evidence for dual or triple GLP-1-based polyagonists compared with placebo in MASLD or MASH Reasons for downgrading: ¹ Risk of bias due to incomplete post-treatment biopsy assessments and missing-data handling. ² Risk of bias due to modified intention-to-treat analyses or inclusion only of participants with available post-baseline MRI-PDFF data. ³ Serious inconsistency because of substantial heterogeneity and imprecision due to a wide confidence interval. ⁴ Serious inconsistency and imprecision because the confidence interval included both little or no effect and a clinically relevant increase in adverse events. ⁵ Risk of bias and serious imprecision because of few events and wide confidence intervals compatible with both benefit and clinically important harm. CI, confidence interval; GRADE, Grading of Recommendations Assessment, Development and Evaluation; MASH, metabolic dysfunction-associated steatohepatitis; MASLD, metabolic dysfunction-associated steatotic liver disease; MD, mean difference; MRI-PDFF, magnetic resonance imaging–proton density fat fraction; RCT, randomized controlled trial; RR, risk ratio. ⨁⨁⨁◯, moderate certainty; ⨁⨁◯◯, low certainty.

Outcome	No. of studies (participants)	Effect estimate (95% CI)	Heterogeneity (I²)	Certainty of evidence (GRADE)	Interpretation
MASH resolution or histological improvement without worsening of fibrosis	3 RCTs (n = 695)	RR 3.32 (2.28–4.84)	20%	⨁⨁⨁◯ Moderate¹	Dual or triple GLP-1-based polyagonists probably increase the probability of MASH resolution or histological improvement without worsening of fibrosis
Fibrosis improvement without worsening of MASH	3 RCTs (n = 695)	RR 1.49 (1.15–1.94)	0%	⨁⨁⨁◯ Moderate¹	Dual or triple GLP-1-based polyagonists probably increase the probability of fibrosis improvement without worsening of MASH
Relative percentage change in liver fat content	3 RCTs (n = 485)	MD −44.60 percentage points (−51.17 to −38.04)	0%	⨁⨁⨁◯ Moderate²	Dual or triple GLP-1-based polyagonists probably produce a substantial reduction in liver fat content
≥30% relative reduction in liver fat content by MRI-PDFF	3 RCTs (n = 570)	RR 4.71 (3.04–7.30)	22%	⨁⨁⨁◯ Moderate²	Dual or triple GLP-1-based polyagonists probably increase the probability of achieving a clinically meaningful imaging response
Percentage change in body weight	2 RCTs (n = 310)	MD −9.14 percentage points (−17.38 to −0.91)	98%	⨁⨁◯◯ Low³	Dual or triple GLP-1-based polyagonists may reduce body weight, but the magnitude of the effect is uncertain because of marked between-study heterogeneity
Any adverse event	4 RCTs (n = 770)	RR 1.17 (0.98–1.39)	79%	⨁⨁◯◯ Low⁴	The effect on the overall incidence of adverse events is uncertain; the confidence interval includes little or no difference and a potentially clinically relevant increase
Serious adverse events	5 RCTs (n = 864)	RR 1.05 (0.54–2.01)	0%	⨁⨁◯◯ Low⁵	The effect on serious adverse events is uncertain because the estimate is imprecise and compatible with both potential benefit and clinically important harm
Adverse events leading to treatment discontinuation	5 RCTs (n = 864)	RR 1.89 (0.60–5.88)	41%	⨁⨁◯◯ Low⁵	The effect on treatment discontinuation due to adverse events is uncertain because the confidence interval is wide and includes both little or no difference and a substantial increase

Discussion

Principal Findings

This systematic review and meta-analysis included six randomized, double-blind, placebo-controlled trials evaluating dual or triple GLP-1-based polyagonists in adults with MASLD or MASH. The pooled results demonstrated significant benefits across histological and imaging-based liver outcomes. Compared with placebo, polyagonist therapy was associated with a more than threefold higher likelihood of MASH resolution or histological improvement without worsening of fibrosis and a 49% higher likelihood of at least one-stage fibrosis improvement without worsening of MASH.

Imaging outcomes were similarly favorable. Dual or triple polyagonists produced a mean relative reduction in liver fat content of approximately 45 percentage points compared with placebo and increased nearly fivefold the probability of achieving a relative liver fat reduction of at least 30% by MRI-PDFF. A significant reduction in body weight was also observed, although this estimate was based on only two studies and showed considerable heterogeneity.

The main safety signal was gastrointestinal intolerance. Nausea, diarrhea, and vomiting occurred significantly more frequently with polyagonists. However, no statistically significant differences were observed in serious adverse events or adverse events leading to treatment discontinuation. Overall, these results suggest that dual and triple GLP-1-based polyagonists provide meaningful hepatic and metabolic benefits, although tolerability and the limited amount of long-term evidence remain important considerations.

Interpretation of Histological and Imaging Outcomes

The histological findings are particularly relevant because fibrosis stage is the strongest histological determinant of liver-related complications and mortality in MASLD [8]. Current EASL-EASD-EASO (European Association for the Study of the Liver/European Association for the Study of Diabetes/European Association for the Study of Obesity), AASLD, AACE, AGA, and ADA guidance emphasizes identification of patients with clinically significant fibrosis, sustained weight reduction, and comprehensive treatment of obesity, diabetes, dyslipidemia, and cardiovascular risk [2-6]. The present findings support the potential value of treatments that address both hepatic disease activity and its systemic metabolic drivers.

The pooled effect for MASH resolution or improvement without worsening of fibrosis was large and statistically consistent. However, the endpoint was not identical across all trials. Tirzepatide and pemvidutide primarily evaluated MASH resolution, whereas the survodutide trial evaluated histological MASH improvement [15,16,18]. The combined estimate should therefore be interpreted as evidence of improvement in steatohepatitis activity rather than as a uniform estimate of complete MASH resolution.

The significant pooled improvement in fibrosis is also encouraging. Tirzepatide and survodutide showed favorable fibrosis outcomes after 52 and 48 weeks, respectively, whereas pemvidutide significantly improved MASH resolution at 24 weeks but did not independently demonstrate a statistically significant fibrosis benefit at that time point [15,16,18]. This difference may reflect treatment duration because fibrosis remodeling generally occurs more slowly than reductions in steatosis and inflammatory activity.

These results place polyagonists within a rapidly evolving therapeutic landscape. Resmetirom demonstrated significant improvements in MASH resolution and fibrosis in the phase 3 MAESTRO-NASH trial [11]. In addition, the phase 3 ESSENCE study demonstrated that semaglutide improved steatohepatitis resolution and fibrosis in patients with MASH and moderate-to-advanced fibrosis [26]. Dual and triple polyagonists may provide an additional advantage through greater effects on body weight, insulin resistance, and hepatic fat, but direct comparative trials are required before superiority over selective GLP-1 receptor agonists or liver-directed therapies can be established.

The MRI-PDFF findings reinforce the metabolic and hepatic activity of this drug class. A relative reduction of at least 30% in MRI-PDFF has been associated with a higher probability of histological response in MASH [27,28]. Therefore, the nearly fivefold higher probability of achieving this threshold may be clinically meaningful and may help identify an early treatment response.

Nevertheless, MRI-PDFF measures hepatic triglyceride content and does not directly assess ballooning, inflammation, or fibrosis. A marked reduction in liver fat should not be considered equivalent to MASH resolution or fibrosis regression. Imaging-based MASLD trials and biopsy-confirmed MASH trials should therefore be viewed as complementary but clinically distinct sources of evidence.

Metabolic and Mechanistic Implications

The observed improvements are biologically plausible given the multifactorial pathogenesis of MASLD and MASH. Excess adiposity, adipose tissue dysfunction, insulin resistance, increased hepatic free fatty acid delivery, de novo lipogenesis, lipotoxicity, inflammatory processes and hepatic stellate cell activation promote ongoing liver damage [9].

GLP-1 receptor activation decreases appetite, promotes weight loss, and improves glycemic control and insulin sensitivity [12]. GIP receptor agonism may provide additional metabolic and adipose tissue effects, whereas glucagon receptor activation may increase energy expenditure, hepatic lipid oxidation, and mobilization of intrahepatic fat. Combined receptor activation may therefore reduce liver fat through both weight-dependent and partially weight-independent mechanisms.

The significant reduction in body weight observed in this meta-analysis is clinically relevant because sustained weight loss is associated with MASH resolution and fibrosis regression [10]. However, the body weight analysis showed very high heterogeneity. Differences in receptor combinations, relative receptor potency, dose, titration, treatment duration, and baseline obesity probably contributed to the variability.

The included agents should consequently not be considered interchangeable. Tirzepatide activates GIP and GLP-1 receptors, survodutide, pemvidutide, and cotadutide activate GLP-1 and glucagon receptors, and retatrutide activates GIP, GLP-1, and glucagon receptors [15-21]. These pharmacological differences may influence the balance among weight loss, liver fat reduction, glycemic control, histological benefit, and adverse effects.

Safety and Clinical Relevance

Gastrointestinal events were the principal tolerability limitation. Nausea was approximately three times more frequent and diarrhea almost twice as frequent with polyagonists. Vomiting also showed a large relative increase, although the estimate was influenced by very low or zero event rates in some placebo groups and had a wide confidence interval.

Despite these gastrointestinal effects, the pooled analyses did not identify a statistically significant increase in serious adverse events or treatment discontinuation. These findings are reassuring but should not be interpreted as definitive evidence of long-term safety. The included studies were predominantly phase 2 trials, sample sizes were modest, and follow-up ranged from 12 to 52 weeks.

Overall adverse events showed substantial heterogeneity, likely related to differences in doses, escalation protocols, treatment duration, and adverse-event reporting. Slow dose escalation may reduce gastrointestinal intolerance, but the optimal balance between receptor activity, efficacy, and tolerability remains uncertain.

From a clinical perspective, these agents are attractive because MASLD is a multisystem metabolic disease rather than an isolated hepatic disorder. Current guidelines recommend management of obesity, diabetes, cardiovascular risk, and liver fibrosis as interconnected therapeutic priorities [2-6]. A treatment capable of improving hepatic histology while also producing weight loss and metabolic benefit could be particularly useful in patients with obesity or type 2 diabetes and at-risk MASH.

However, the findings do not support a uniform class recommendation. Most of the evaluated agents remain investigational for MASH, and treatment decisions should continue to reflect approved indications, fibrosis stage, metabolic comorbidities, safety, availability, and cost. Direct comparisons with semaglutide, resmetirom, and other emerging therapies will be necessary to define the position of polyagonists in future treatment algorithms.

Strengths and Limitations

The principal strength of this review is its targeted inclusion of randomized placebo-controlled trials evaluating dual and triple GLP-1-based polyagonists. Histological, imaging-based, metabolic, and safety outcomes were analyzed separately, avoiding the assumption that reductions in liver fat are equivalent to histological improvement. Risk of bias was assessed using RoB 2, and certainty of evidence was evaluated using GRADE.

Several limitations should be considered. First, only six trials were included, with two to five studies contributing to each outcome. This limited the ability to conduct reliable subgroup, dose-response, sensitivity, or publication-bias analyses.

Second, the interventions were pharmacologically heterogeneous. Pooling different receptor combinations and doses provides a class-level estimate but may obscure clinically important differences among individual agents.

Third, study populations varied considerably. Some trials enrolled patients with biopsy-confirmed MASH and fibrosis, whereas others selected participants with obesity and elevated liver fat by MRI-PDFF. Treatment duration also ranged from 12 to 52 weeks, which is particularly relevant because short-term trials may detect liver fat reduction but not fibrosis remodeling.

Fourth, several trials had incomplete post-treatment biopsy or MRI assessments or used modified intention-to-treat populations, resulting in some concerns regarding missing outcome data. Most studies were also industry-sponsored, emphasizing the importance of independent confirmation.

Fifth, the available safety data should be interpreted cautiously. Because the included trials had relatively small sample sizes and limited treatment durations, rare adverse events and long-term safety signals may have been missed. Therefore, the absence of a statistically significant increase in serious adverse events or adverse events leading to treatment discontinuation should not be interpreted as definitive evidence of long-term safety.

Finally, Embase and some additional bibliographic databases were unavailable. Although PubMed, CENTRAL, Google Scholar, ScienceDirect, and reference-list screening were used, incompletely indexed or unpublished studies might have been missed.

Future Research

Future phase 3 trials should include larger populations, longer treatment periods, standardized histological definitions, central blinded pathology review, and paired biopsy and MRI-PDFF assessments. Reporting should include absolute as well as relative adverse-event rates, dose-specific outcomes, and standardized definitions of treatment discontinuation.

Further research should determine whether early MRI-PDFF response predicts subsequent histological improvement and whether the magnitude of weight loss mediates MASH resolution or fibrosis regression. Drug-specific and dose-specific analyses will also be required to identify the receptor profile with the most favorable benefit-risk balance.

Direct comparative and combination-treatment trials are likely to become increasingly important. Polyagonists could potentially be compared or combined with liver-directed treatments such as resmetirom, particularly in patients with both advanced metabolic dysfunction and clinically significant fibrosis. Ultimately, trials should assess liver-related and cardiovascular clinical outcomes rather than relying exclusively on surrogate histological or imaging endpoints.

## Conclusions

Dual and triple GLP-1-based polyagonists significantly improved histological and imaging-based outcomes in adults with MASLD or MASH. Compared with placebo, these agents increased the likelihood of MASH resolution or improvement without worsening of fibrosis, improved the probability of fibrosis regression without worsening of MASH, substantially reduced liver fat content, and increased the proportion of patients achieving a clinically meaningful MRI-PDFF response. Polyagonist therapy was also associated with weight reduction, although this result showed considerable heterogeneity. Gastrointestinal adverse events, particularly nausea, diarrhea, and vomiting, were more frequent, but no statistically significant increase was observed in serious adverse events or treatment discontinuation.

These findings support dual and triple GLP-1-based polyagonists as promising therapies capable of targeting both hepatic disease activity and underlying cardiometabolic dysfunction. Nevertheless, the limited number of trials, pharmacological heterogeneity, short follow-up, and lack of clinical outcome data prevent definitive conclusions regarding individual agents or long-term benefit. Larger phase 3 trials and direct comparative studies are required to determine their role in future MASLD and MASH treatment algorithms.

## Appendices

Appendix 1

**Table 3:** Database-specific search strategies used in the systematic review Searches were conducted from database inception to May 19, 2026. Database-specific syntax was adapted as required. Because ScienceDirect limited the number of Boolean operators that could be used in a single query, separate searches were performed for each intervention. Google Scholar results were sorted by relevance, and the first 100 records were screened. No language restrictions were applied during the initial search. CENTRAL: Cochrane Central Register of Controlled Trials; MASH: metabolic dysfunction-associated steatohepatitis; MASLD: metabolic dysfunction-associated steatotic liver disease; NAFLD: nonalcoholic fatty liver disease; NASH: nonalcoholic steatohepatitis.

Database/source	Search strategy	Filters/limits	Date searched
PubMed/MEDLINE	(MASLD[Title/Abstract] OR MASH[Title/Abstract] OR NAFLD[Title/Abstract] OR NASH[Title/Abstract] OR "metabolic dysfunction-associated steatotic liver disease"[Title/Abstract] OR "metabolic dysfunction associated steatotic liver disease"[Title/Abstract] OR "metabolic dysfunction-associated steatohepatitis"[Title/Abstract] OR "metabolic dysfunction associated steatohepatitis"[Title/Abstract] OR "nonalcoholic fatty liver disease"[Title/Abstract] OR "non-alcoholic fatty liver disease"[Title/Abstract] OR "nonalcoholic steatohepatitis"[Title/Abstract] OR "non-alcoholic steatohepatitis"[Title/Abstract] OR "fatty liver"[MeSH Terms]) AND (tirzepatide[Title/Abstract] OR survodutide[Title/Abstract] OR pemvidutide[Title/Abstract] OR efinopegdutide[Title/Abstract] OR cotadutide[Title/Abstract] OR retatrutide[Title/Abstract] OR "GLP-1/glucagon"[Title/Abstract] OR "GLP-1 glucagon"[Title/Abstract] OR "glucagon-like peptide-1/glucagon"[Title/Abstract] OR "glucagon-like peptide 1 glucagon"[Title/Abstract] OR "GLP-1/GIP"[Title/Abstract] OR "GIP/GLP-1"[Title/Abstract] OR "GLP-1/GCG"[Title/Abstract] OR "GIP/GLP-1/glucagon"[Title/Abstract] OR "dual agonist"[Title/Abstract] OR "dual receptor agonist"[Title/Abstract] OR "dual incretin"[Title/Abstract] OR "triple agonist"[Title/Abstract] OR "triple receptor agonist"[Title/Abstract] OR polyagonist[Title/Abstract] OR coagonist[Title/Abstract] OR "co-agonist"[Title/Abstract]) AND (randomized controlled trial[Publication Type] OR controlled clinical trial[Publication Type] OR randomized[Title/Abstract] OR randomised[Title/Abstract] OR placebo[Title/Abstract] OR trial[Title]) NOT (animals[MeSH Terms] NOT humans[MeSH Terms]).	No language restrictions; randomized or controlled clinical studies involving adults with MASLD, MASH, NAFLD, or NASH were considered during screening	May 19, 2026
Cochrane Central Register of Controlled Trials (CENTRAL)	(MASLD OR MASH OR NAFLD OR NASH OR "metabolic dysfunction-associated steatotic liver disease" OR "metabolic dysfunction associated steatotic liver disease" OR "metabolic dysfunction-associated steatohepatitis" OR "metabolic dysfunction associated steatohepatitis" OR "nonalcoholic fatty liver disease" OR "non-alcoholic fatty liver disease" OR "nonalcoholic steatohepatitis" OR "non-alcoholic steatohepatitis" OR "fatty liver" OR steatohepatitis) AND (tirzepatide OR survodutide OR pemvidutide OR efinopegdutide OR cotadutide OR retatrutide OR "GLP-1 glucagon" OR "GLP-1 GIP" OR "GIP GLP-1 glucagon" OR "dual agonist" OR "dual receptor agonist" OR "triple agonist" OR "triple receptor agonist" OR polyagonist OR coagonist OR "co-agonist") AND (randomized OR randomised OR placebo OR trial).	Trials database; no language restrictions	May 19, 2026
ScienceDirect	(drug name) AND (MASLD OR MASH OR NAFLD OR NASH OR "fatty liver" OR steatohepatitis). Each search was repeated using tirzepatide, survodutide, pemvidutide, efinopegdutide, cotadutide, and retatrutide as the intervention term. Results were restricted to research articles and were screened by title and abstract for potential eligibility.	Research articles; searches conducted separately for each drug	May 19, 2026
Google Scholar	(MASLD OR MASH OR NAFLD OR NASH OR "metabolic dysfunction-associated steatotic liver disease" OR "metabolic dysfunction-associated steatohepatitis" OR "nonalcoholic fatty liver disease" OR "non-alcoholic fatty liver disease" OR "nonalcoholic steatohepatitis" OR "non-alcoholic steatohepatitis" OR "fatty liver" OR steatohepatitis) AND (tirzepatide OR LY3298176 OR survodutide OR BI456906 OR pemvidutide OR ALT-801 OR efinopegdutide OR MK-6024 OR cotadutide OR MEDI0382 OR retatrutide OR LY3437943 OR efocipegtrutide OR HM15211 OR "dual agonist" OR "dual receptor agonist" OR "triple agonist" OR "triple receptor agonist" OR polyagonist OR coagonist OR "co-agonist") AND (randomized OR randomised OR placebo OR trial).	First 100 records sorted by relevance	May 19, 2026
Manual search	Reference lists of included studies and relevant reviews	Additional potentially eligible randomized controlled trials and reports	May 19, 2026

## References

[1] Rinella ME, Lazarus JV, Ratziu V (2023). A multisociety Delphi consensus statement on new fatty liver disease nomenclature. Hepatology.

[2] European Association for the Study of the Liver (EASL) (2024). EASL-EASD-EASO clinical practice guidelines on the management of metabolic dysfunction-associated steatotic liver disease (MASLD). J Hepatol.

[3] Rinella ME, Neuschwander-Tetri BA, Siddiqui MS (2023). AASLD practice guidance on the clinical assessment and management of nonalcoholic fatty liver disease. Hepatology.

[4] Cusi K, Isaacs S, Barb D (2022). American Association of Clinical Endocrinology clinical practice guideline for the diagnosis and management of nonalcoholic fatty liver disease in primary care and endocrinology clinical settings: co-sponsored by the American Association for the Studies. Endocr Pract.

[5] Kanwal F, Shubrook JH, Adams LA (2021). Clinical care pathway for the risk stratification and management of patients with nonalcoholic fatty liver disease. Gastroenterology.

[6] Cusi K, Abdelmalek MF, Apovian CM (2025). Metabolic dysfunction-associated steatotic liver disease (MASLD) in people with diabetes: the need for screening and early intervention. A consensus report of the American Diabetes Association. Diabetes Care.

[7] Riazi K, Azhari H, Charette JH (2022). The prevalence and incidence of NAFLD worldwide: a systematic review and meta-analysis. Lancet Gastroenterol Hepatol.

[8] Taylor RS, Taylor RJ, Bayliss S (2020). Association between fibrosis stage and outcomes of patients with nonalcoholic fatty liver disease: a systematic review and meta-analysis. Gastroenterology.

[9] Loomba R, Friedman SL, Shulman GI (2021). Mechanisms and disease consequences of nonalcoholic fatty liver disease. Cell.

[10] Vilar-Gomez E, Martinez-Perez Y, Calzadilla-Bertot L (2015). Weight loss through lifestyle modification significantly reduces features of nonalcoholic steatohepatitis. Gastroenterology.

[11] Harrison SA, Bedossa P, Guy CD (2024). A phase 3, randomized, controlled trial of resmetirom in NASH with liver fibrosis. N Engl J Med.

[12] Drucker DJ (2018). Mechanisms of action and therapeutic application of glucagon-like peptide-1. Cell Metab.

[13] Armstrong MJ, Gaunt P, Aithal GP (2016). Liraglutide safety and efficacy in patients with non-alcoholic steatohepatitis (LEAN): a multicentre, double-blind, randomised, placebo-controlled phase 2 study. Lancet.

[14] Newsome PN, Buchholtz K, Cusi K (2021). A placebo-controlled trial of subcutaneous semaglutide in nonalcoholic steatohepatitis. N Engl J Med.

[15] Loomba R, Hartman ML, Lawitz EJ (2024). Tirzepatide for metabolic dysfunction-associated steatohepatitis with liver fibrosis. N Engl J Med.

[16] Sanyal AJ, Bedossa P, Fraessdorf M (2024). A phase 2 randomized trial of survodutide in MASH and fibrosis. N Engl J Med.

[17] Harrison SA, Browne SK, Suschak JJ (2025). Effect of pemvidutide, a GLP-1/glucagon dual receptor agonist, on MASLD: a randomized, double-blind, placebo-controlled study. J Hepatol.

[18] Noureddin M, Harrison SA, Loomba R (2025). Safety and efficacy of weekly pemvidutide versus placebo for metabolic dysfunction-associated steatohepatitis (IMPACT): 24-week results from a multicentre, randomised, double-blind, phase 2b study. Lancet.

[19] Romero-Gómez M, Lawitz E, Shankar RR (2023). A phase IIa active-comparator-controlled study to evaluate the efficacy and safety of efinopegdutide in patients with non-alcoholic fatty liver disease. J Hepatol.

[20] Sanyal AJ, Kaplan LM, Frias JP (2024). Triple hormone receptor agonist retatrutide for metabolic dysfunction-associated steatotic liver disease: a randomized phase 2a trial. Nat Med.

[21] Shankar SS, Daniels SJ, Robertson D (2024). Safety and efficacy of novel incretin co-agonist cotadutide in biopsy-proven noncirrhotic MASH with fibrosis. Clin Gastroenterol Hepatol.

[22] Mantovani A, Petracca G, Beatrice G, Csermely A, Lonardo A, Targher G (2021). Glucagon-like peptide-1 receptor agonists for treatment of nonalcoholic fatty liver disease and nonalcoholic steatohepatitis: an updated meta-analysis of randomized controlled trials. Metabolites.

[23] Page MJ, McKenzie JE, Bossuyt PM (2021). The PRISMA 2020 statement: an updated guideline for reporting systematic reviews. BMJ.

[24] Higgins JPT, Thomas J, Chandler J (2019). Cochrane Handbook for Systematic Reviews of Interventions. 2nd Edition.

[25] Sterne JA, Savović J, Page MJ (2019). RoB 2: a revised tool for assessing risk of bias in randomised trials. BMJ.

[26] Sanyal AJ, Newsome PN, Kliers I (2025). Phase 3 trial of semaglutide in metabolic dysfunction-associated steatohepatitis. N Engl J Med.

[27] Loomba R, Neuschwander-Tetri BA, Sanyal A (2020). Multicenter validation of association between decline in MRI-PDFF and histologic response in NASH. Hepatology.

[28] Stine JG, Munaganuru N, Barnard A (2021). Change in MRI-PDFF and histologic response in patients with nonalcoholic steatohepatitis: a systematic review and meta-analysis. Clin Gastroenterol Hepatol.

